# The effects of health insurance and physical exercise participation on life satisfaction of older people in China—Based on CHNS panel data from 2006 to 2015

**DOI:** 10.3389/fpubh.2022.858191

**Published:** 2022-08-26

**Authors:** Lin Luo, Xiaojin Zeng, Xiangfei Wang

**Affiliations:** ^1^College of Physical Education, Guizhou Normal University, Guiyang, China; ^2^Basic Education Research Center, Southwest University, Chongqing, China; ^3^East China Normal University—Xuhui Education Group Postdoctoral Workstation, Shanghai, China; ^4^Research Institute of Sports Science, Wuhan Sports University, Wuhan, China

**Keywords:** older people, active aging, physical exercise, health insurance, CHNS

## Abstract

**Background:**

In China, the problem of aging population has become more and more serious. The factors influencing life satisfaction of older people are becoming a significant issue. This study explores the effects of health insurance and physical exercise on life satisfaction of older people in China.

**Method:**

This study used an unbalanced panel dataset (*n* = 6,393, T = 4) of older adults aged 60–80 years from the 2006 to 2015 China Health and Nutrition Survey (CHNS). A panel ordered logistic regression model was developed to examine the effects of health insurance and physical exercise on older people' life satisfaction. Mediation tests were used to examine the mediating role of physical exercise in the effect of health insurance on life satisfaction of older people.

**Result:**

Life satisfaction of older people was positively associated with participation in health insurance (OR = 1.439) and physical exercise (OR = 1.033). Participation in government health insurance, urban employee health insurance (UEBMI), new rural cooperative health insurance (NRCMI), and other commercial health insurance all have positive effects on life satisfaction of older people. Physical exercise plays a masking role in the effect of health insurance on life satisfaction of older people.

**Conclusion:**

Participation in health insurance and physical exercise are important means to promote life satisfaction among older people. Physical exercise affects the impact of health insurance on older people's life satisfaction.

## Introduction

Over the past three decades, China has experienced rapid economic growth and tremendous demographic change (1, 2). By the end of 2017, China's population aged over 60 reached 241 million, accounting for 17.3% of the total population (3), with a serious trend of population aging (4). Actively coping with population aging is not only related to the quality of life of the elderly, but will also have far-reaching effects on the whole society and economy.

The World Health Organization (WHO) adopted the term active aging in the late 1990s and played an important role in its rapid spread (5). The WHO envisages active aging as a broad process of optimizing opportunities for health, participation, and security to improve the quality of life of people as they age (6). The WHO states that the goal of active aging is to improve the quality of life of older people (7). Life satisfaction is not only a subjective perception of older people's living conditions, but also an important indicator of their quality of life (8). Therefore, a more detailed and in-depth study of the factors influencing the life satisfaction of older people will help to further improve the quality of life of older people and has important practical significance in promoting the formulation of public policies on active aging.

## Literature review

### Factors influencing the life satisfaction of older people

In addition to reflecting an individual's life evaluation of emotions, happiness, and subjective wellbeing, life satisfaction indirectly reflects an individual's perception and judgement of the difference between their expectations and the reality of their quality of life (9, 10). Changes in older people's life satisfaction are a complex and dynamic process that may be the result of a combination of subtle individual and environmental changes over time (6). As the process of population aging continues, the factors influencing older people's life satisfaction have received increasing attention.

Research findings are inconsistent in terms of the impact of gender on life satisfaction among older adults. Li and Liu and Du and Wang found that older men had lower life satisfaction than women (11, 12). Akifusa et al. found that older men were more satisfied with their lives than women (13). Massey et al. found no significant relationship between gender and life satisfaction in older people (14). In terms of the effect of age on life satisfaction among older people, Angelini et al. and Liu et al. found a positive relationship between age and life satisfaction among older people (15, 16), but Li et al. only observed this relationship in older people aged 80 years and above (11). George et al. found little age variability in life satisfaction among older adults (17). Although Li et al. and Du and Wang found that life satisfaction was higher among urban older people than rural older people in the early years (11, 12). However, the gap in life satisfaction between the urban and rural elderly populations is gradually narrowing as people's income levels in rural areas increase (12). In terms of the effect of spouse status on older adults' life satisfaction, Massey et al. and Liu et al. found that older adults with a spouse had higher life satisfaction (12, 14, 16). However, Li et al. found that spousal status did not significantly affect older adults' life satisfaction (11). Li et al. and Du and Wang found that educational attainment had a significant positive impact on older people's life satisfaction (11, 12). However, Knight et al. found that educational attainment *per se* did not affect older people's life satisfaction. It is the other material and spiritual income gained through education that is an important factor in older people's life satisfaction (18). In terms of the relationship between income and life satisfaction, Wu and Chen found a “threshold effect” between income levels and older people's life satisfaction, with the effect of income on life satisfaction decreasing after a certain level is reached (19). Tavares' study found that income was a driver of life satisfaction among older people (20). In terms of the impact of health status on life satisfaction in older adults, Celso et al. and Celik et al. found a statistically significant relationship between health status and life satisfaction in older adults (21, 22). Jung et al. found that older adults with chronic conditions had significantly lower life satisfaction compared to those without chronic conditions (23). Wiesmann et al. found that the higher the number of chronic conditions, the lower the life satisfaction scores of older people (24). There are also environmental factors that may have an impact on the life satisfaction of older people. There are also environmental factors that may have an impact on the life satisfaction of older people. For example, Proto et al. found that regional GDP also influences people's life satisfaction (25). Rajani et al. (26) found that higher GDP was associated with higher life satisfaction scores. Dingemans et al., however, found that in regions with poorer GDP, it was continuing to work after retirement that had a positive effect on older people's life satisfaction (27). In summary, existing studies have found that gender, age, educational attainment, income, Chronic disease, GDP, and work after retirement may all have a significant impact on older people's life satisfaction.

### Health insurance and life satisfaction of older people

Chinese scholars have used data from the China Longevity Health Influence Survey (CLHLS), the China Health and Aging Tracking Survey (CHARLS), the China Sample Survey on the Living Conditions of the Elderly in Urban and Rural Areas (SSAPUR), the China Elderly Social Tracking Survey (CLASS), the Sixth Population Census, or survey data collected independently by researchers to conduct an in-depth study of older people's Life satisfaction has been explored in depth. Easterlin et al., Walker, and Li et al., found that although economic development has led to a significant increase in life satisfaction among Chinese residents, the growth trend of their life satisfaction has been declining (1, 5, 9). Yu et al., Appleton et al., and Knight et al., found that changes in life satisfaction among Chinese residents were mainly related to two factors: the transition of the Chinese economy (from a planned to a free market economy) accompanied by an increase in income inequality and the disintegration of the traditional social security system (28–30). Wang et al. found that despite the increase in household income among urban residents, income inequality and fear of unemployment reduced their life satisfaction (31). Ng et al. found that the change in the social security system from an employer-based system to an insurance-based system led to uncertainty about unemployment, which in turn reduced people's life satisfaction (32). Wang et al. and WTO found that this inequality and insecurity was particularly present among vulnerable groups, such as the elderly and economically economically disadvantaged people (31, 33).

Older people are often particularly concerned about the disintegration of the traditional social security system and the high cost of health care in a market-oriented healthcare system. As older people age, they are at high risk of deteriorating health status. For example, Cai and Wang found that over 100 million people aged 60 and over suffered from at least one chronic disease (e.g., stroke, heart disease). Approximately 5 million older people suffer from mental health problems (e.g., depression and dementia) (34). Health insurance is therefore particularly important for older people with chronic diseases and other illnesses, and can go some way to safeguarding their quality of life (33). Yip and Hsiao point out that the cost of healthcare services has been rising under the influence of economic restructuring and the marketisation of the healthcare delivery system (35). Data from the World Bank shows that more and more elderly people are falling into poverty due to high out-of-pocket health care costs (36). Li and Liu found that in 2008 ~31% of rural older people reported that they were unable to access adequate health services (11). Sun et al. found that 14% of urban older self-reported illnesses were the main cause of financial insecurity (37).

In response to this situation, the Chinese government has initiated a series of comprehensive health insurance system reforms to provide basic health insurance services to all citizens, including the elderly. In 2003 and 2007, the New Rural Cooperative Medical Scheme (NCMS) and the Urban Residents Basic Health Insurance (URBMI) for rural residents were set up. Together with the Government Health Insurance (GMI) and the Urban Employees' Health Insurance (UEMI) for staff of government agencies or state institutions, they form the country's universal health insurance system. Older people in particular also benefit from the government's expanded health insurance services. In 2012, Sun et al. found that 98.4% of older Chinese reported being covered by some kind of health insurance (37).

Although the coverage and scope of different health insurance schemes vary, studies by Keng et al. and Tran et al. found that access to health insurance can counter the insecurity associated with health shocks and uncertainty about health expenditures. This is particularly important for older people who are at greater risk of deteriorating health and financial status (38, 39). Rao and Gao found that people with health insurance tended to make greater use of health services and reported better health and wellbeing (40). Wu and Li found that health insurance could help older people feel secure and was positively associated with their physical and mental health (41). Kim and Koh found that lack of health insurance had a negative impact on the subjective wellbeing of the general population after controlling for individual self-assessed health status and other socioeconomic factors (42). While the aim of universal health coverage is to ensure equal access to health services, Liao et al. found that universal health coverage also improved subjective wellbeing among older people, particularly older women (43). A previous study by Yang and Hanewald reported that the life satisfaction of middle-aged and older Chinese (aged 45–60 years) was not related to whether they had health insurance, but rather to the type of health insurance they chose. Compared to residents who participated in government health insurance, those who participated in urban employees' health insurance, urban residents' health insurance and the New Agricultural Cooperative had lower life satisfaction scores of 0.155, 0.106, and 0.112 standard deviations, respectively (44).

### Physical exercise and life satisfaction of older people

Physical exercise is an active and healthy lifestyle. Physical exercise can have physiological, psychological and social effects. Reyes et al. and Leitner and Leitner found a positive relationship between Physical exercise and personal life satisfaction (45, 46). Sardeli et al. and Papi and Cheraghi found that greater acquisition of physical ability can promote individuals to have a sense of competence and increased motivation to make motivation to make decisions. This in turn promotes intrinsic motivation through the achievement of practical satisfaction (47, 48). Physical exercise is a self-selected experience with a sense of freedom and intrinsic motivation. In addition, Physical exercise may have additional health benefits for older adults. Hao et al. and Dai and Yao found that Physical exercise increased muscle mass, reduced the risk of fat and obesity, improved balance and reduced fear of falling in older adults (49, 50). Papi and Cheraghi and Liao et al. found that older adults' cognitive abilities, such as short-term and long-term memory, verbal reasoning and risk of cognitive impairment, were also positively influenced by Physical exercise (48, 51). However, previous literature suggests that not all Physical exercise contributes to life satisfaction in older adults. Life satisfaction may depend on the type of exercise or the duration of the exercise. For example, Mudrák et al. found a significant relationship between participation in Physical exercise and life satisfaction. Participants who achieved the recommended level of Physical exercise (moderate and/or vigorous exercise) were more satisfied with their lives (52). In a Swedish study of 176 community-dwelling older adults, Hao et al. found that older participants in the moderate and vigorous Physical exercise groups had higher life satisfaction than those in the low Physical exercise group (49). However, there are studies with different perspectives, such as Dai and Yao, which found no direct relationship between Physical exercise and life satisfaction in older adults. Physical exercise only indirectly influences older people's life satisfaction through self-efficacy and the support of friends (50).

Numerous scholars have conducted a large number of studies on life satisfaction among the elderly and its influencing factors, from which it can be concluded that the influencing factors of life satisfaction among the elderly may be complex, involving various aspects of the individual, family and society. Existing research has begun to actively focus on the relationship between health insurance and life satisfaction among older people. However, the relationship between health insurance participation and life satisfaction among older people over 60 years of age in China is not yet clear. Also, further confirmation is needed as to whether physical exercise also has a positive effect on enhancing life satisfaction among older people in China. In terms of data, scholars have mostly used census data, sample survey data or tracking survey data for their analyses, however, most studies have used cross-sectional data for their analyses. The limited sample size of survey data other than census makes it difficult to achieve panel data studies with large samples. In terms of research methods, descriptive statistical analysis, logistic regression models and other generalized linear models or multiple linear regression analysis are mostly used. Although significant factors affecting the life satisfaction of older people can be identified, it is difficult to control for variables that do not change over time and to identify the significant influencing factors that cause changes in life satisfaction of older people. In view of this, this study uses the theory of “active coping theory” as the theoretical support to explore the effects of health insurance coverage and physical exercise participation on older people's life satisfaction based on previous studies. At the same time, based on the China Health and Nutrition Survey (CHNS) data from 2006 to 2015, a random effects ordered logistic regression model is used to analyse the large sample panel data, which can control for individual heterogeneity and identify potential factors affecting changes in life satisfaction of the elderly, and then determine the effects and mechanisms of health insurance participation and physical exercise participation on life satisfaction of the elderly. This study will provide a reference for the formulation of public policies on active aging.

## Research hypothesis

In 1997, Aspinwall and Taylor introduced the well-known “active coping theory,” which considers active coping as efforts made before a potentially stressful event occurs (53). Parada and Verlhiac define coping as activities that master, tolerate, reduce, or minimize environmental or psychological demands (54). There are important differences between active coping and anticipatory coping for stressful events. Active coping refers to the need for skills and activities that are different from, and potentially more successful than, coping with an existing stressor prior to coping and anticipatory coping. Lee et al. argue that active coping theory can provide an adequate theoretical context for Physical exercise and health insurance participation among older adults (55). Older adults have potential health and economic stressors due to their declining physical and economic status. Therefore, Salamene et al. suggest that physical exercise and health insurance participation can be seen as a positive coping behavior and a positive action to prevent future health stress (56). Based on active coping theory and combined with the findings of previous studies, this study proposes the following hypotheses.

*H1*: Participation in health insurance is positively associated with life satisfaction among older adults.

*H2*: Physical exercise participation is positively associated with life satisfaction among older adults.

In addition, Salamene et al. found that older adults' coping resources and current health status can significantly influence active coping styles (56). Physical and mental health of older adults found by Cao and Lu (57) and Lawless et al. (58) can influence physical exercise behavior. Hyun and Ku, Vannini et al., and Aldwin et al. found that coping resources such as income (59), education (60), and other factors that may influence stressors (61), such as chronic illness, may also influence older adults' physical exercise behavior. Blanco-Molina et al. found that health insurance was one of the factors that may influence one of the factors of stressors (62). Therefore, health insurance may also influence physical exercise behavior of older adults. For example, de Boer et al. found that societies with a higher proportion of sports club members had lower average health insurance consumption (63). Cheah et al. found that exercisers who participated in sporting activities were more likely to have health insurance (64), which may of course be related to the potentially higher socioeconomic status of the participants. Zhang et al. found in values for older adults aged 55–75 years that those with more health insurance had less physical exercise behavior compared to the group without health insurance (65). While it is not possible to determine whether participation in health insurance has a positive effect on physical exercise among older adults based on the available studies, it can be hypothesized that health insurance can influence individuals' physical exercise behavior. Therefore, physical exercise may be a mediating variable in the mechanism of the effect of health insurance participation on life satisfaction of older adults. To this end, the following hypotheses were formulated for this study.

*H3*: The impact of physical exercise-mediated health insurance participation on older people's life satisfaction.

**Figure 1:**
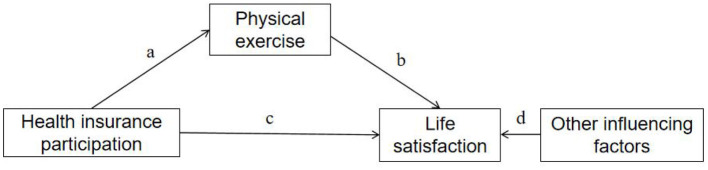
Theoretical assumptions model.

Based on theoretical assumptions, a theoretical model was developed for this study (see Figure 1). The model suggests that health insurance participation can directly affect older people's life satisfaction (path a). Physical exercise can directly affect older people's life satisfaction (path b). And participation in health insurance can indirectly affect older people's life satisfaction through physical exercise (path c). Other factors such as gender, age, education level, health status, urban/rural category, spousal status, annual income, and regional economic development may also influence older people's life satisfaction (path d).

## Research subjects and methods

### Research sample

This study used original data from the China Health and Nutrition Tracking Survey (CHNS) conducted by the Population Center of the University of Carolina at Chapel Hill and the Chinese Center for Disease Control and Prevention since 1989 (66). The CHNS is longitudinal and includes 1989, 1991, 1993, 1997, 2000, 2004, 2006, 2009, 2011, and 2015, with a total of 10 waves covering nine provinces (Heilongjiang, Liaoning, Jiangsu, Shandong, Henan, Henan, Hubei, Hunan, Guangxi, and Guizhou) and three autonomous cities (Beijing, Shanghai, and Chongqing). The questionnaires used in each wave were kept as similar as possible.

In order to achieve the aims of the study, the research team used three criteria to limit the sample for the study. Firstly, the CHNS survey team has only been studying people's physical exercise since 2004 and their life satisfaction since 2006. Therefore, this study aimed to use data from 2006 and onwards for the analysis of the study. Secondly, given the timing of retirement and the actual physical exercise of most Chinese residents, the age range of the study population was restricted to 60–80 years. Thirdly, those with limitations in activities of daily living were excluded. After considering these inclusion and exclusion criteria and after data cleaning, this study resulted in an unbalanced short panel dataset (*n* = 6393, T = 4).

### Dependent and independent variables

CHNS used the question “How do you feel about your life now?” to survey the life satisfaction of older people (67). This study defines the sum of various health-related insurance policies purchased by residents as the main explanatory variable health insurance for this study. The CHNS survey on individual health insurance covers various types of health insurance, including Urban Employees' Health Insurance (UEBMI), New Rural Cooperative Health Insurance (NRCMI), Urban Residents' Basic Health Insurance (URBMI), and the purchase of various commercial health insurance policies. The Urban Employees' Health Insurance (68) is a medical benefit provided to employees of Chinese companies, but the amount of contribution varies from company to company. In addition to the company's contribution, individuals are also required to pay a portion of their own costs. The New Rural Cooperative Health Insurance Scheme is a highly subsidized voluntary health insurance scheme (69). The Basic Health Insurance for Urban Residents is a subsidized voluntary public health insurance scheme introduced by the Chinese government for urban residents who are not formally employed (70). There are some differences in the categorization of health-related insurance surveys conducted by CHNS in different years. And with reference to the relevant category settings of the 2015 questionnaire, this study combined the insurance categories of health insurance for the elderly into three categories: employee health insurance (EMI), resident health insurance (RMI) (both NPS and resident) and commercial health insurance (CHI).Thus, the “health insurance” variable in the study is actually a combination of RMI, EMI, and CHI. In addition, a focus on annual individual health insurance premiums (including all types of health insurance premiums paid by individuals) was added. The CHNS survey covers questions on physical exercise participation, including how much time residents spend on martial arts (kung fu, etc.), gymnastics, dance, acrobatics, athletics (running), swimming, football, basketball, tennis, badminton, volleyball, and other sports. It asks all older people “How much time (minutes) do you spend on average each day doing the following physical activities, Monday to Friday or Saturday to Sunday? In this study, the time spent by older people on physical exercise during the week was aggregated and named as physical exercise (minutes/week).

### Covariates

In this study, gender, age, education level, spouse status, place of residence, chronic illness, annual income, GDP per capita, and work were selected as Covariates. With reference to Cheng's study (68), GDP per capita in this study was used as the source of data from the China Urban Statistical Yearbook published by the National Bureau of Statistics in the current year. This study also controls for the dummy variables of province and occupation of the study population.

### Description of variables

Annual income, GDP per capita, physical exercise, and health insurance premiums were continuous variables in this study and their logarithmic form was used when included in the analytical model. The remaining variables are categorical, with life satisfaction being an ordered categorical variable. The results of the descriptive analysis of the study variables are shown in Table 1.

**Table 1:** Descriptive statistics for variables (*n* = 6,393).

**Variable**	**Description**	***Mean (SD)***
Health insurance participation	EMI/RMI/CHI (No = 0, Yes = 1)	0.874 (0.276)
Health insurance premium	The total annual payment of individual healthl insurance (RMB). Take the logarithm for analysis	175.203 (0.802)
EMI	Government Health Insurance (GMI)/UEBMI(No = 0, Yes = 1)	0.240 (0.406)
RMI	NRCMI/URBMI(No = 0, Yes = 1)	0.596 (0.446)
CHI	Various commercial health insurance/commercial medical insurance (No = 0, Yes = 1)	0.021 (0.125)
Life satisfaction	How do you think your life is now? (1 = very bad ~ 5 = very good)	3.595 (0.763)
Gender	Male = 0, Female = 1	0.527 (0.499)
Age (year)	60–64 = 1; 65–69 = 2; 70–74 = 3; 75–80 = 4	2.140 (1.050)
Education attainment	Primary school and below = 1; Junior high school = 2; High School/Higher Vocational School/Secondary School = 3; College and above = 4	1.602 (0.908)
Spouse status	None (single, divorced) = 0, Yes (married, separated, widowed) = 1	0.983 (0.123)
Residence	City = 0, Rural = 1	0.505 (0.484)
Chronic disease	Whether you have high blood pressure, diabetes, myocardial infarction, stroke, tumor, asthma? (No = 0, Yes = 1, total number of diseases)	0.251 (0.408)
Annual income	Annual salary income + annual bonus + annual pension + annual income from other sources (RMB). Take the logarithm for analysis	12350.240 (3285.940)
GDP per capita	GDP per capita (RMB). Take the logarithm for analysis	41422.860 (5787.601)
Physical exercise (PE)	Average weekly physical exercise time (min/week)	157.580 (394.511)
Job	Are you still working? No = 0, Yes = 1	0.223 (0.363)

### Statistical methods

This study used STATA 16.0 software for statistical processing of the survey data. Logistic regression models were used in analyzing the effects of physical exercise and health insurance participation on the life satisfaction of older people. CHNS is an unbalanced panel. This study conducted a joint F-test for time effects and found no significant time effect (*p* = 0.419) on the change in life satisfaction of older people. A Sobel test was used to analyse the mediating effect of participation in health insurance on the life satisfaction of older people.

### Ethics, approval, and informed consent

We used the public dataset from CHNS official website (https://www.cpc.unc.edu/projects/china). Therefore, the Academic Committee of the School of Physical Education of Guizhou Normal University waived the requirement for ethical approval. CHNS provides interviewees with guarantees of privacy and confidentiality. All participants provided written informed consent. Detailed information about the research design is on its official website.

## Result

### Factors influencing the life satisfaction of older people

Before conducting an ordered logistic regression model analysis, in order for the model to fit better, the relationship between the independent and dependent variables needs to be tested first and unnecessary variables eliminated. The stepwise regression analysis revealed no significant relationship between gender, spouse status and life satisfaction of the elderly, so these two variables were excluded. To prevent endogeneity, the variables needed to be tested for independence from each other and the absence of multicollinearity. Therefore, the study continued with the regression analysis of the remaining independent and dependent variables, and all independent variables had VIF < 10 and 1/VIF > 0.1, suggesting that there was no serious co-linearity between the independent variables. The study included all independent variables, except gender and spouse status, in the subsequent analysis model. This study used a random effects ordered logistic regression model to analyse the factors influencing life satisfaction among older people, with the output being the Odds Ratio (OR) (see Table 2). Model (1) is a regression of age, education attainment, residence, chronic disease, log annual income, log GDP, and job on life satisfaction. Model (2) is a regression incorporating log health insurance premiums based on model (1). Model (3) is a regression that incorporates health insurance participation based on model (1). Model (4) is a regression based on model (1) that continues to include different health insurance participation. Model (5) is a regression based on model (3) that continues to include log physical exercise. All regression models control for dummy variables for individual's previous occupation and province.

**Table 2:** Results of randomized ordered logistic regression of factors influencing older people's life satisfaction (*n* = 6,393).

**Variable**	**Health insurance participation**				
	**Model (1)**	**Model (2)**	**Model (3)**	**Model (4)**	**Model (5)**
**Age** ^**a**^					
65–69	0.910^**^ (0.042)	0.911^**^ (0.042)	0.915^**^ (0.042)	0.918^**^ (0.042)	0.913^**^ (0.042)
70–74	0.859^***^ (0.046)	0.859^***^ (0.046)	0.862^***^ (0.046)	0.859^***^ (0.046)	0.864^***^ (0.046)
75–80	0.840^***^ (0.050)	0.841^***^ (0.051)	0.849^***^ (0.051)	0.843^***^ (0.051)	0.858^***^ (0.051)
**Education attainment** ^**b**^					
Middle school	1.382^***^ (0.078)	1.382^***^ (0.075)	1.365^***^ (0.074)	1.343^***^ (0.074)	1.330^***^ (0.074)
High/Higher Vocational/Secondary School	1.865^***^ (0.132)	1.862^***^ (0.124)	1.828^***^ (0.122)	1.756^***^ (0.122)	1.736^***^ (0.122)
College and above	1.912^***^ (0.202)	1.917^***^ (0.185)	1.872^***^ (0.180)	1.775^***^ (0.180)	1.749^***^ (0.180)
Residence^c^	0.709^***^ (0.036)	0.707^**^ (0.039)	0.711^***^ (0.044)	0.758^***^ (0.044)	0.745^***^ (0.044)
Chronic disease	0.763^***^ (0.041)	0.763^***^ (0.041)	0.764^***^ (0.041)	0.759^***^ (0.041)	0.770^***^ (0.041)
Ln annual income	1.009^**^ (0.003)	1.009^**^ (0.003)	1.009^**^ (0.003)	1.009^**^ (0.003)	1.009^**^ (0.003)
Ln GDP	1.488^***^ (0.068)	1.476^***^ (0.068)	1.330^***^ (0.069)	1.323^***^ (0.069)	1.185^***^ (0.069)
Job^d^	3.350^**^ (1.684)	3.354^**^ (1.839)	3.223^**^ (1.847)	3.195^**^ (1.767)	3.531^**^ (1.767)
Ln Health insurance premium		1.001 (0.005)			
Health insurance participation^e^			1.404^***^ (0.094)		1.439^**^ (0.097)
EMI^f^				1.506^***^ (0.108)	
RMI^i^				1.208^***^ (0.083)	
CHI^j^				1.581^***^ (0.207)	
Ln PE					1.033^***^ (0.004)
**Dummy province variable**	**Control**				
Cut1	−1.069 (0.519)	−1.156 (0.544)	−1.952 (0.607)	−2.081 (0.617)	−3.156 (0.638)
Cut2	1.041 (0.514)	−0.955 (0.538)	0.161 (0.602)	0.313 (0.612)	−1.042 (0.633)
Cut3	3.929 (0.516)	23.842 (0.540)	3.049 (0.603)	2.921 (0.613)	1.845 (0.633)
Cut4	6.112 (0.519)	6.025 (0.542)	5.231 (0.605)	5.108 (0.614)	4.031 (0.635)
Sigma_u	0.772 (0.069)	0.772 (0.069)	0.759 (0.068)	0.765 (0.068)	0.746 (0.068)
Wald test value	1061.91	1062.06	1088.21	1098.31	1133.55
Chibar2	228.54	228.56	222.73	224.53	216.68

The values outside and inside the brackets are the OR and standard errors, respectively.

^**^, and ^***^ indicate significance at the 5%, and 1% levels, respectively.

Reference groups: a for 60–64 group, b for Primary school and below group, c for City group, d for no job group, e for no health insurance participation, f for no EMI group, i for no RMI group, j for no CMI group.

From the regression results of the five models, age, education attainment, residence, chronic disease, log annual income, log GDP, job, health insurance participation, EMI, RMI, CMI, and log physical exercise all had significant effects on life satisfaction of the elderly, indicating that these factors play an important role in terms of life satisfaction of the elderly. However, the relationship between log health insurance premiums and life satisfaction of the elderly was not significant.

Specifically, the results from Model 1 reveal that there is a negative relationship between age and life satisfaction of older people. Compared to the reference group of 60–64 year old, the OR of increased life satisfaction for 65–69, 70–74, and 75–80 year olds decreased by 9.0, 14.1, and 16% respectively. There was a positive relationship between education attainment and life satisfaction of older people. Compared to the primary school and below reference group, the OR for increased life satisfaction increased by 38.2, 86.5, and 91.2% for Middle school, High/Higher Vocational/Secondary School, College and above, respectively. The OR of increased life satisfaction for rural older adults decreased by 29.4% compared to the city reference group. There was a negative relationship between the number of Chronic disease and life satisfaction of older people. For each unit increase in chronic disease, the OR of increased life satisfaction for older people decreased by 23.7%. There was a positive relationship between log annual income and life satisfaction among older people. For each unit increase in log annual income, the OR for increase in life satisfaction of older people increased by 0.9%. There was a positive relationship between log GDP and older people's life satisfaction. For each unit increase in log GDP, the OR for increased life satisfaction of older people increased by 48.8%. Compared to the no job reference group, the OR of increase in life satisfaction for older people with a job increased by 235%.

The results from model 2 show that the relationship between the independent variables and life satisfaction of older people remains significant after the inclusion of log health insurance premiums, but the effect of log health insurance premiums on health insurance for older people is not significant.

The results of model 3 showed that the relationship between the independent variable and life satisfaction of the elderly remained significant after the inclusion of health insurance premiums, and that there was a significant effect of health insurance premiums on the life satisfaction of the elderly. There was a 40.4% increase in the likelihood of increased life satisfaction for those with health insurance participation compared to those without health insurance participation. The results of Model 4 showed that participation in EMI, RMI, and CMI all had a positive effect on the increase in life satisfaction of older people. Compared to uninsured older adults, the ORs of increased life satisfaction for EMI, RMI, and CMI were 50.6, 20.8, and 58.1%, respectively. The results from Model 5 revealed that the relationship between the independent variables and life satisfaction of older adults remained significant after the inclusion of log physical exercise, and log physical exercise had a significant effect on life satisfaction of older adults. Each unit increase in log physical exercise was associated with a 3.3% increase in life satisfaction OR for older people.

The results of Models 1, 3, and 5 show that health insurance participation and log physical exercise moderate the effects of age and educational attainment on older people's life satisfaction to some extent. Logarithmic physical exercise is more likely to increase life satisfaction among rural older people than health insurance participation. Logarithmic physical exercise also improves the life satisfaction of older people with chronic diseases. Health insurance participation moderates the effect of job on older people's satisfaction and increases the life satisfaction of older people who are no job. Both health insurance participation and log physical exercise moderate the effect of GDP on older people's life satisfaction and reduce the effect of regional economic development on older people's life satisfaction.

### Intermediary test

The results of the regression analysis of this study (Table 2) showed that older people's participation in physical exercise and health insurance had a positive impact on their life satisfaction. However, the pathways through which health insurance participation affects older people's life satisfaction merit further research. A review analysis of past studies suggests that health insurance participation may affect older people's life satisfaction by either promoting or discouraging their physical exercise. Therefore, in this paper, regular physical activity was chosen as a mediating variable to examine the potential mechanisms of health insurance participation on older people's life satisfaction. Models (6)—(8) in Table 3 report the regression results of the mediation test for the effect of health insurance participation on older people's life satisfaction. Model (6) reflects the results of the test without the inclusion of mediating variables. It can be seen that, similar to the previous section, there is a significant positive effect of health insurance participation on older people's life satisfaction. The results of model (7) show that participation in health insurance has a significant negative effect on log physical exercise among older people. Model (8) reflects the results of the test after the inclusion of mediating variables, showing that the effect of health insurance participation on older people's life satisfaction remains significantly positive after the inclusion of log physical exercise, and the regression coefficient becomes larger. The results suggest that the variable log physical exercise masks to some extent the effect of health insurance participation on older people's life satisfaction. Controlling for the variable log physical exercise significantly increases the effect of health insurance participation on life satisfaction. This suggests that some older adults with health insurance may reduce their physical exercise time, thereby affecting their life satisfaction.

**Table 3:** Intermediary test results.

	**Model (6)**	**Model (7)**	**Model (8)**
	**Life satisfaction**	**Ln PE**	**Life satisfaction**
Health insurance participation	0.163^***^ (0.027)	−0.235^*^ (0.136)	0.166^***^ (0.027)
Ln PE			0.015^***^ (0.002)
**Control variable**	**Control**		
_cons	2.305 (0.249)	−45.113 (1.247)	2.960 (0.262)
R-squared	0.109	0.241	0.114

The control variables are individual characteristics, regional macroeconomic characteristics, and provincial dummy variables.

Values outside and in parentheses are estimated coefficients and standard errors, respectively.

^*^, and ^***^ indicate significance at the 10 and 1% levels.

## Discussion

In this study research hypotheses H1, H2, and H3 were all validated by the empirical data. The regression results showed that after controlling for age, educational attainment, residence, chronic disease, annual income, GDP, job, occupation, and province, there was a significant positive effect on older people's life satisfaction, regardless of the type of health insurance participation. Consistent with the findings of Rao and Gao (40) and Wu and Li (41), but different from the findings of Yang and Hanewald (44). Yang and Hanewald concluded that older adults' life satisfaction was not related to whether they participated in health insurance, but rather to the type of health insurance they chose. Although the ORs of EMI, RMI, and CMI on the increase of life satisfaction in old age were not the same in this study, simply having health insurance could have a significant positive effect on the life satisfaction of older adults. The main reason for analyzing the difference between the results of this study and those of Yang et al. may be related to the age of the study population in that study, which was 45–60 years old (44). Those aged 45–60 were more likely to be involved in the workforce and to receive work-plus-health insurance benefits than those aged 60 and beyond. Also in this study, no significant relationship was found between health insurance premiums and life satisfaction among older adults. A previous study by Liao et al. found that the impact of health insurance premiums on older people's life satisfaction varied by gender, with women being more affected than men (43).

This study found that health insurance participation and physical activity moderated the effect of age on life satisfaction among older people to some extent. As older people age, their physical functioning will continue to decline at any time. At the same time, physical exercise for older people decreases as some of the physical activities they used to do are gradually reduced or replaced by other tasks due to changes in lifestyle. In addition, physical exercise for older people not only enhances physical fitness, prevents disease and improves immunity, but also increases their social participation and social interaction (33), which in turn improves the impact of age or older people's life satisfaction. As age increases, older people's health risks increase, and increased health risks can increase the level of health care consumption among older people. Without the help of health insurance, this may result in an increased financial burden for older people's families. Therefore, participation in health insurance can increase older people's sense of security against uncertain future health risks and financial risks (65).

This study found that health insurance participation and physical exercise moderated to some extent the effect of educational attainment on life satisfaction among older adults.“ Data collected by the “Gallup World Poll” shows a positive relationship between education and life satisfaction (71). Although the components of life satisfaction are complex, research suggests that social and emotional skills play a role in determining life satisfaction. Social and emotional skills come primarily from schooling, but also from the family and cultural environment (72). In countries with greater educational differences, higher educational attainment increases the proportion of adults who self-report life satisfaction (73). In countries with greater educational disparities, higher educational attainment increases the proportion of adults who self-report life satisfaction (74). Physical exercise is beneficial in promoting health equity (75). This study found that physical exercise reduces the significant effect of education on older adults' life satisfaction, which may be related to the fact that physical exercise helps to reduce health disparities between older adults with different levels of education, which in turn reduces the effect of educational attainment on older adults' life satisfaction. Health insurance participation reduced the significant effect of education on older adults' life satisfaction, which may be related to the fact that health insurance helps to reduce health disparities and health coverage disparities among older adults with different levels of education, increasing the accessibility of health care for older adults with different levels of education, and thus reducing the effect of education on older adults' life satisfaction. Sun et al. used data from a nationwide panel and confirmed improved health outcomes for residents who utilized health insurance coverage provided to urban residents (37). Su et al. found that older adults with health insurance participation had higher subjective wellbeing than those without health insurance participation. Health insurance participation helped to reduce health inequities associated with education and thus increased life satisfaction among older adults with different levels of education (75).

This study found that health insurance participation and physical exercise helped to reduce the impact of urban-rural differences on older people's life satisfaction. Looking at the urban and rural typologies, life satisfaction was significantly lower among rural older people than urban older people. A previous study reported that urban older people with chronic diseases had higher life satisfaction than rural older people with chronic diseases (76). There are significant differences between urban and rural areas in terms of lifestyle, household economic level and social security, and these factors may contribute to the differences in life satisfaction between urban and rural older people (77). Health insurance participation increases the level of health care coverage for older people in both urban and rural areas. Health insurance is particularly important for older people, which basically guarantees their quality of life (78). With socio-economic development, the health needs of older people have escalated and physical exercise can improve not only their health but also their lifestyles. Physical exercise can reduce the impact of urban-rural differences on older people's life satisfaction and help to improve the life satisfaction of rural older people.

Physical exercise can also improve the life satisfaction of older people with chronic illnesses, as the decline in physical function and health risks are more pronounced with age than when they were younger. This confirms the adage that “life is about exercise.” Health insurance participation moderates the impact of work on older people's satisfaction and increases the life satisfaction of older people who are not working. Individuals who are employed are also likely to have higher health insurance contributions, which may potentially reduce their sense of security against uncertain future health and financial risks, thereby increasing their life satisfaction. The “Easterlin paradox” suggests that the relationship between the level of economic development and life satisfaction in a country or region is not a positive one (79). Xiang and Yao found a positive correlation between life satisfaction and household income in China, while there was no significant correlation between life satisfaction and regional economic development at the macro level (76). In contrast, this study found that GDP of the elderly significantly affects the life satisfaction of the elderly. The original intention of our government to set up different types of health insurance was to promote equity in health protection for different residents (79). However, due to regional differences in the level of economic development, the accessibility of health insurance for older people in economically developed regions may be better than that in less economically developed regions. Health insurance plays an important role in reducing the impact of differences in economic development on the life satisfaction of older people. Physical activity and health insurance both have a significant impact on life satisfaction among older people. Therefore, encouraging older people to take part in health insurance and physical exercise is one of the important means to achieve active aging strategies.

Cheah et al. study reported that individuals who participated in physical exercise were more likely to have health insurance (64). However, Zhang et al. the higher the amount of health insurance purchase, the less physical exercise behavior (65). The results of this study found that the effect of health insurance participation on older adults' life satisfaction remained significantly positive after the inclusion of regular physical exercise, and the regression coefficient became larger, thus indicating that physical exercise masked the effect of health insurance participation on older adults' life satisfaction to some extent, and that controlling for the variable of physical exercise significantly amplified the effect of health insurance participation on life satisfaction. The results of the study suggest that in the process of promoting active aging and healthy aging in China, special attention should be paid to those older people who have reduced their physical activity behavior as a result of their participation in health insurance.

This study also has some limitations, which should be taken into account when interpreting the results of this study. First, health insurance and physical exercise in this study were derived from self-reports by participants. Consequently, the results may have been affected by false positives, which may have weakened the associations observed in the study. Second, although many factors influencing life satisfaction in older adults have been documented in the literature, this study was unable to control for them as covariates in the study because they were not in the original study and these data were not available for this study. This study does not use instrumental variables and other test analysis methods for the endogeneity of variables, and hopes to further improve in future research. Finally, due to the second-hand public data used in this study, which is affected by the sampling method and sample distribution of the CHNS database, the relevant conclusions of this study need to be further verified in other large national samples in the future.

## Conclusion

Participation in health insurance and physical exercise are important means to promote life satisfaction among older people. Physical exercise affects the impact of health insurance on older people's life satisfaction.

## Data availability statement

The datasets presented in this study can be found in online repositories. The names of the repository/repositories and accession number(s) can be found in the article/supplementary material.

## Ethics statement

The studies involving human participants were reviewed and approved by CHNS provides interviewees with guarantees of privacy and confidentiality. All participants provided written informed consent. Detailed information about the research design is on its official website. The patients/participants provided their written informed consent to participate in this study.

## Author contributions

LL conceived the study and performed the data analysis and interpretation. LL, XZ, and XW drafted the manuscript. LL and XZ participated in the refinement of the manuscript. All authors have read and approved the final manuscript.

## Funding

This research used data from the China Health and Nutrition Survey (CHNS). We thank the National Institute of Nutrition and Food Safety, China Center for Disease Control and Prevention, Carolina Population Center, the University of North Carolina at Chapel Hill, the NIH (R01-HD30880, DK056350, and R01-HD38700) and the Fogarty International Center, NIH for financial support for the CHNS data collection and analysis files from 1989 to 2006, and both parties and the China–Japan Friendship Hospital, Ministry of Health for support for the CHNS 2009 and future surveys. Funding for this research came from the East China Normal University-Xuhui Postdoctoral Workstation Fund (No. 2019001), the Guizhou Provincial Department of Education Youth Growth Project Fund [Qianjiao He KY (2021) 291], and the Guizhou Province Education Planning Fund Project (2021A058).

## Conflict of interest

The authors declare that the research was conducted in the absence of any commercial or financial relationships that could be construed as a potential conflict of interest.

## Publisher's note

All claims expressed in this article are solely those of the authors and do not necessarily represent those of their affiliated organizations, or those of the publisher, the editors and the reviewers. Any product that may be evaluated in this article, or claim that may be made by its manufacturer, is not guaranteed or endorsed by the publisher.

## References

[1] Easterlin RA Wang F Wang S. Growth and happiness in China, 1990–2015. In: A modern guide to the economics of happiness. Edward Elgar Publishing (2021). p. 129–161. 10.4337/9781788978767.00017

[2] Beard JR Officer AM Cassels AK. The world report on ageing and health. Gerontologist. (2016) 56:S163–6. 10.1093/geront/gnw037 26994257

[3] Han Y Fu JP. Policy supply of elderly care services in China: evolution, governance framework, and future directions. Lanzhou Acad J. (2020) 9:187–98.

[4] Pei CH. The new goal of building a new system of a higher-level open economy - a little experience from studying ”The CPC Central Committee's Suggestions on Formulating the Fourteenth Five-Year Plan for National Economic and Social Development and the Long-term Goals for 2035“. Res Ref. (2020) 24:89–93. 10.16110/j.cnki.issn2095-3151.2020.24.009

[5] Walker A. A strategy for active ageing. Int Soc Secur Rev. (2002) 55:121–39. 10.1111/1468-246X.00118

[6] World Health Organization. Active Ageing: A Policy Framework (No. WHO/NMH/NPH/02. 8) (2002). 12040973

[7] World Health Organization. Report of the World Health Organization: active ageing: a policy framework. Aging Male. (2002) 5:1–37. 10.1080/tam.5.1.1.37 12040973

[8] Pahlevan SS Amiri M Allen KA Sharif NH Khoshnavay FF Hatef MY. Attachment: the mediating role of hope, religiosity, and life satisfaction in older adults. Health Qual Life Outcomes. (2021) 19:57. 10.1186/s12955-021-01695-y 33588858 PMC7885200

[9] Li Y Wu Q Liu C Kang Z Xie X Yin H. Catastrophic health expenditure and rural household impoverishment in China: what role does the new cooperative health insurance scheme play? PLoS ONE. (2014) 9:e93253. 10.1371/journal.pone.0093253 24714605 PMC3979676

[10] Tavakoly Sany SB Aman N Jangi F Lael-Monfared E Tehrani H Jafar A. Quality of life and life satisfaction among university students: exploring, subjective norms, general health, optimism, and attitude as potential mediators. J Am Coll Health. (2021) 9:1–8. 10.1080/07448481.2021.1920597 34242514

[11] Li JX Liu BZ. Differences and changes in life satisfaction of urban and rural elderly population: based on CLHLS project survey data. Xuehai. (2015) 1:101–10.

[12] Du P Wang B. How internet use affects life satisfaction of Chinese elderly? Popul Res. (2020) 44:3–17. Available online at: http://www.cqvip.com/qk/95654x/202004/7102482214.html

[13] Akifusa S Soh I Ansai T Hamasaki T Takata Y Yohida A. Relationship of number of remaining teeth to health-related quality of life in communit-dwelling elderly. Gerodontology. (2005) 22:91–7. 10.1111/j.1741-2358.2005.00059.x 15934350

[14] Massey B Edwards AV Musikanski L. Life satisfaction, affect, and belonging in older adults. Appl Res Qual Life. (2021) 16:1205–19. 10.1007/s11482-019-09804-2

[15] Angelini V Cavapozzi D Corazzini L Paccagnella O. Age, health and life satisfaction among older Europeans. Soc Indic Res. (2012) 105:293–308. 10.1007/s11205-011-9882-x 22207782 PMC3228960

[16] Liu BB Liu XM. Research on the influence of interpersonal relationships on life satisfaction of the elderly under the background of active aging. Soc Secur Res. (2021) 5:1–11.

[17] George LK Okun MA Landerman R. Age as a moderator of the determinants of life satisfaction. Res Aging. (1985) 7:209–33. 10.1177/0164027585007002004 4059636

[18] Knight J Lina S Gunatilaka R. Subjective wellbeing and its determinants in rural China. China Econ Rev. (2009) 20:635–49. 10.1016/j.chieco.2008.09.00327349854

[19] Wu LM Chen HX. Construction of structural equation model of income and happiness index-taking small towns in Zhejiang province as an example. China Rural Econ. (2010) 11:63–74.

[20] Tavares AI. Health and life satisfaction factors of Portuguese older adults. Arch Gerontol Geriatr. (2022) 99:104600. 10.1016/j.archger.2021.104600 34883397

[21] Celso BG Ebener DJ Burkhead EJ. Humor coping, health status, and life satisfaction among older adults residing in assisted living facilities. Aging Ment Health. (2003) 7:438–45. 10.1080/13607860310001594691 14578005

[22] Celik SS Celik Y Hikmet N Khan MM. Factors affecting life satisfaction of older adults in Turkey. Int J Aging Hum Dev. (2018) 87:392–414. 10.1177/0091415017740677 29124946

[23] Jung MS Muntaner C Choi MK. Factors related to perceived life satisfaction among the elderly in South Korea. J Prev Med Public Health. (2010) 43:292–300. 10.3961/jpmph.2010.43.4.292 20689355

[24] Wiesmann U Hannich HJ. The contribution of resistance resources and sense of coherence to life satisfaction in older age. J Happiness Stud. (2013) 14:911–28. 10.1007/s10902-012-9361-3

[25] Proto E Rustichini A. A reassessment of the relationship between GDP and life satisfaction. PLoS ONE. (2013) 8:e79358. 10.1371/journal.pone.0079358 24312179 PMC3842267

[26] Rajani NB Skianis V Filippidis FT. Association of environmental and sociodemographic factors with life satisfaction in 27 European countries. BMC Public Health. (2019) 19:534. 10.1186/s12889-019-6886-y 31077185 PMC6509815

[27] Dingemans E Henkens K. Working after retirement and life satisfaction: cross-national comparative research in Europe. Res Aging. (2019) 41:648–69. 10.1177/0164027519830610 30782077

[28] Yu L Yan Z Yang X Wang L Zhao Y. Impact of social changes and birth cohort on subjective well-being in Chinese older adults: a cross-temporal meta-analysis, 1990-2010. Soc Indic Res. (2016) 126:795–812. 10.1007/s11205-015-0907-8

[29] Appleton S Song L. Life satisfaction in urban China: components and determinants. World Dev. (2008) 36:2325–40. 10.1016/j.worlddev.2008.04.00932795293

[30] Knight J Gunatilaka R. Does economic growth raise happiness in China? Oxf Dev Stud. (2011) 39:1–24. 10.1080/13600818.2010.551006

[31] Wang P Pan J Luo Z. The impact of income inequality on individual happiness: evidence from China. Soc Indic Res. (2015) 121:413–35. 10.1007/s11205-014-0651-5

[32] Ng ST Tey NP Asadullah MN. What matters for life satisfaction among the oldest-old? Evidence from China. PLoS ONE. (2017) 12:e0171799. 10.1371/journal.pone.0171799 28187153 PMC5302476

[33] World Health Organization. China Country Assessment Report on Ageing Health. (2015). p. 34.

[34] Cai F Wang M. Growth and structural changes in employment in transition China. J Comp Econ. (2010) 38:71–81. 10.1016/j.jce.2009.10.00612178548

[35] Yip WCM Hsiao WC. Non-evidence-based policy: how effective is China's new cooperative medical scheme in reducing medical impoverishment? In: Health Care Policy in East Asia: A World Scientific Reference: Volume 1: Health Care System Reform and Policy Research in China. (2020). p. 85–105. 10.1142/9789813236134_000519019519

[36] World Bank. Live Long and Prosper: Aging in East Asia and Pacific. The World Bank (2015).

[37] Sun J Deng S Xiong X Tang S. Equity in access to healthcare among the urban elderly in China: does health insurance matter? Int J Health Plann Manag. (2014) 29:e127–44. 10.1002/hpm.2227 25028751

[38] Keng SH Wu SY. Living happily ever after? The effect of Taiwan's National Health Insurance on the happiness of the elderly. J Happiness Stud. (2014) 15:783–808. 10.1007/s10902-013-9449-4

[39] Tran NLT Wassmer RW Lascher EL. The health insurance and life satisfaction connection. J Happiness Stud. (2017) 18:409–26. 10.1007/s10902-016-9729-x

[40] Rao K Gao J. Research on National Health Services-An Analysis Report of the Second National Health Service Survey in 1998. Beijing: Ministry of Health, PRC. (1999). p. 75.

[41] Wu X Li J. Economic growth, income inequality and subjective well-being: evidence from China. Popul Stud Cent Res Rep. (2013). Available online at: http://hdl.handle.net/1783.1/85827

[42] Kim S Koh K. Health insurance and subjective well-being: evidence from two healthcare reforms in the United States. Health Econ. (2021) 31:233–49. 10.1002/hec.4448 34727396

[43] Liao PA Chang HH Sun LC. National Health Insurance program and life satisfaction of the elderly. Aging Ment Health. (2012) 16:983–92. 10.1080/13607863.2012.692765 22681404

[44] Yang S Hanewald K. Life satisfaction of middle-aged and older Chinese: the role of health and health insurance. Soc Indic Res. (2022) 160:601–24. 10.1007/s11205-020-02390-z

[45] Reyes Fernández B Fleig L Godinho CA Montenegro ME Knoll N Schwarzer R. Action control bridges the planning-behaviour gap: a longitudinal study on physical exercise in young adults. Psychol Health. (2015) 30:911–23. 10.1080/08870446.2015.1006222 25587901

[46] Leitner MJ Leitner SF. Leisure in Later Life. Binghamton, NY: Haworth Press (1996).

[47] Sardeli AV Griffth GJ Dos Santos MVMA Ito MSR Chacon-Mikahil MPT. The effects of exercise training on hypertensive older adults: an umbrella meta-analysis. Hypertens Res. (2021) 44:1434–43. 10.1038/s41440-021-00715-0 34385687

[48] Papi S Cheraghi M. Multiple factors associated with life satisfaction in older adults. Prz Menopauzalny. (2021) 20:65–71. 10.5114/pm.2021.107025 34321983 PMC8297631

[49] Hao W Li J Fu P Zhao D Jing Z Wang Y. Physical frailty and health-related quality of life among Chinese rural older adults: a moderated mediation analysis of physical disability and physical activity. BMJ Open. (2021) 11:e042496. 10.1136/bmjopen-2020-042496 33419914 PMC7799141

[50] Dai Q Yao JX. The relationship between physical exercise and life satisfaction of theelderly: the mediating role of self-efficacy, social support, and self-esteem. J Beijing Sport Univ. (2012) 35:67–72. 10.19582/j.cnki.11-3785/g8.2012.05.015

[51] Liao YH Kao TW Peng TC Chang YW. Gender differences in the association between physical activity and health-related quality of life among community-dwelling elders. Aging Clin Exp Res. (2021) 33:901–8. 10.1007/s40520-020-01597-x 32462499

[52] Mudrák J Slepička P Šiška P. Physical activity and life satisfaction in seniors participating in educational programs. Auc Kinanthropol. (2015) 47:84–95. Avaialble online at: https://karolinum.cz/casopis/auc-kinanthropologica/rocnik-47/cislo-1/clanek-779

[53] Aspinwall LG Taylor SE. A stitch in time: self-regulation and proactive coping. Psychol Bull. (1997) 121:417. 10.1037/0033-2909.121.3.417 9136643

[54] Parada S Verlhiac JF. Growth mindset intervention among French university students, and its articulation with proactive coping strategies. Educ Psychol. (2021) 42:354–374. 10.1080/01443410.2021.1917519

[55] Lee J Bae H Lee E. Influence of successful aging, quality of life, and factors related to potential stressors on older consumers' purchase of private health insurance in South Korea: an empirical study based on proactive coping theory. J Appl Gerontol. (2021) 41:253–61. 10.1177/07334648211002006 33754868

[56] Salamene LC Martins ELM Lucchetti G Lucchetti ALG. Factors associated with successful aging in Brazilian community-dwelling older adults: when physical health is not enough. Geriatr Nurs. (2021) 42:372–8. 10.1016/j.gerinurse.2021.01.009 33571931

[57] Cao Q Lu B. Mediating and moderating effects of loneliness between social support and life satisfaction among empty nesters in China. Curr Psychol. (2021) 40:973–82. 10.1007/s12144-018-0019-0

[58] Lawless MT Tieu M Feo R Kitson AL. Theories of self-care and self-management of long-term conditions by community-dwelling older adults: a systematic review and meta-ethnography. Soc Sci Med. (2021) 287:114393. 10.1016/j.socscimed.2021.114393 34534780

[59] Hyun S Ku X. Proactive coping mediates the relationship between the narcissism phenotypes and psychological health. Soc Behav Pers Int J. (2021) 49:e10477. 10.2224/sbp.10477

[60] Vannini P Gagliardi GP Kuppe M Dossett ML Donovan NJ Gatchel JR. Stress, resilience, and coping strategies in a sample of community-dwelling older adults during COVID-19. J Psychiatr Res. (2021) 138:176–85. 10.1016/j.jpsychires.2021.03.050 33862301 PMC8369528

[61] Aldwin CM Yancura L Lee H. Stress, coping, and aging. In: Schaie KW and Willis S, editors. Handbook of the Psychology of Aging. Academic Press (2021). p. 275–286. 10.1016/B978-0-12-816094-7.00016-7

[62] Blanco-Molina M Pinazo-Hernandis S Montoro-Rodriguez J Tomas JM. Testing a proactive model of successful aging among older adults in Costa Rica and Spain. Int J Aging Hum Dev. (2021) 93:619–35. 10.1177/0091415020974621 33236652

[63] de Boer WI Dekker LH Koning RH Navis GJ Mierau JO. How are lifestyle factors associated with socioeconomic differences in health care costs? Evidence from full population data in the Netherlands. Prev Med. (2020) 130:105929. 10.1016/j.ypmed.2019.105929 31778685

[64] Cheah YK Azahadi M Phang SN Hazilah N. Factors affecting participation decision and amount of physical activity among urban dwellers in Malaysia. Public Health. (2017) 146:84–91. 10.1016/j.puhe.2017.01.009 28404478

[65] Zhang C Lei X Strauss J Zhao Y. Health insurance and health care among the mid-aged and older Chinese: evidence from the national baseline survey of CHARLS. Health Econ. (2017) 26:431–49. 10.1002/hec.3322 26856894 PMC4980285

[66] He K Du S Xun P Sharma S Wang H Zhai F. Consumption of monosodium glutamate in relation to incidence of overweight in Chinese adults: China Health and Nutrition Survey (CHNS). Am J Clin Nutr. (2011) 93:1328–36. 10.3945/ajcn.110.008870 21471280 PMC3095503

[67] Liang Y Lu P. Effect of occupational mobility and health status on life satisfaction of Chinese residents of different occupations: logistic diagonal mobility models analysis of cross-sectional data on eight Chinese provinces. Int J Equity Health. (2014) 13:15. 10.1186/1475-9276-13-15 24506976 PMC3922249

[68] Hesketh T Jun YX Lu L Mei WH. Health status and access to health care of migrant workers in China. Public Health Rep. (2008) 123:189–97. 10.1177/003335490812300211 18457071 PMC2239328

[69] You X Kobayashi Y. The new cooperative medical scheme in China. Health Policy. (2009) 91:1–9. 10.1016/j.healthpol.2008.11.012 19121873

[70] Liu H Zhao Z. Does health insurance matter? Evidence from China's urban resident basic medical insurance. J Comp Econ. (2014) 42:1007–20. 10.1016/j.jce.2014.02.003

[71] Ma ZY Liu SS. The “mirror” and “original image” of China's national well-being Analysis of mutual evidence and QCA adaptation path based on domestic and foreign authoritative databases. Economist. (2019) 10:46–57.

[72] Li HF Chen TY. Social function and subjective well-being of the elderly. Adv Psychol Sci. (2009) 17:759–65. Available online at: https://www.cnki.com.cn/Article/CJFDTotal-XLXD200904018.htm

[73] Yang Rui. How health and life satisfaction are linked with education. J East China Normal Univ. (2017) 35:155–8. 10.16382/j.cnki.1000-5560.2017.05.014

[74] Rodríguez-Pose A Maslauskaite K. Can policy make us happier? Individual characteristics, socio-economic factors and life satisfaction in Central and Eastern Europe. Camb J Reg Econ Soc. (2012) 5:77–96. 10.1093/cjres/rsr038

[75] Su YS Lien D Yao Y. Economic growth and happiness in China: a bayesian multilevel age-period-cohort analysis based on the CGSS data 2005-2015. Int Rev Econ Finance. (2022) 77:191–205. 10.1016/j.iref.2021.09.018

[76] Xiang YH Yao H. Differences in social support between urban and rural elderly and its impact on health status and life satisfaction. J Huazhong Agric Univ. (2016) 6:85–921 + 45. 10.13300/j.cnki.hnwkxb.2016.06.012

[77] Wu Fei Wang J. Relative income and subjective well-being: examining multiple reference groups of migrant workers. Society. (2017) 37:74–105. 10.15992/j.cnki.31-1123/c.2017.02.004

[78] Sidel VW. New lessons from China: equity and economics in rural health care. Am J Public Health. (1993) 83:1665–6. 10.2105/AJPH.83.12.1665 8259789 PMC1694917

[79] Lu N Spencer M Sun Q Lou VW. Family social capital and life satisfaction among older adults living alone in urban China: the moderating role of functional health. Aging Ment Health. (2021) 25:695–702. 10.1080/13607863.2019.1709155 31899943

